# Editorial: Recent advances of epigenetics in crop biotechnology, volume II

**DOI:** 10.3389/fpls.2023.1175539

**Published:** 2023-03-24

**Authors:** Alexandre Berr, Raúl Alvarez-Venegas

**Affiliations:** ^1^ Institut de Biologie Moléculaire des Plantes du CNRS- Université de Strasbourg, Strasbourg, France; ^2^ Genetic Engineering, Center for Research and Advanced Studies, National Polytechnic Institute of Mexico (CINVESTAV), Mexico City, Mexico

**Keywords:** crops, agricultural, biotechnology, chromatin, epigenetics

The study of chromatin modifications and the use of newly developed tools to manipulate different chromatin marks and the epigenome has permeated to all areas of research: from diabetes to neurodegenerative disorders, growth and development, plant-pathogen interactions, and crop biotechnology, to mention but a few.

In some 20 years, scientific publications on epigenetics have increased exponentially, with more than hundred thousand articles published so far on this topic. In the light of this considerable amount of data, we must tackle step by step the intricacies of each epigenetic mechanisms to effectively benefit from their analyses.

Crop performance is one of the long-term threats facing humanity, and an important area of research. One of the key challenges is to unravel epigenetic mechanisms that plants have evolved to cope with biotic and abiotic stresses and develop a stress memory which help them to enhance intra-, inter- and trans-generationally responses against recurring stresses. In the near future, we must be able to edit and manipulate the epigenome as novel directions to drive plant breeding and improve crops. As examples, the generation of new beneficial traits through the creation of novel epialleles or the selection of advantageous epigenetic states seem to be particularly promising.

The goal of this Research Topic was to amalgamate some of the recent advances concerning the role of epigenetics in crop biotechnology, as well as to enhance and promote interactions among high quality researchers from different disciplines. Here we present four research articles, one review and a method that will certainly have an impact on crop epigenetics.

In a first research article, Bvindi et al. demonstrate that two histone lysine methyltransferases, SET Domain Group 33 (SDG33) and SDG34 (*i.e*., orthologs to the H3K36 histone methyltransferase SDG8 from *Arabidopsis*) mediate nitrogen (N) responses of shoots and roots in tomato (*Solanum lycopersicum* L.) in a partially overlapping manner. Interestingly, using transcriptomic profiling of CRISPR edited mutants, authors found that SDG33 and SDG34 control N-responsive gene regulatory networks in an organ-specific manner, suggesting that histone methylation is an epigenetic mechanism involved in N-mediated root plasticity. In addition, SDG33 and SDG34 are involved in maintaining the chlorophyll a/b ratio in leaves when the N level changes. Finally, because *SDG8* in *Arabidopsis* functions as a regulatory hub between N and pathogen responses, authors speculated whether it is the same for SDG33 and SDG34 in tomato.

In a second research article, Han et al. deliver very intriguing results as to the impact of the linker histone H1 on DNA methylation and gene regulation at imprinted loci. As a first step, authors shown, in *Arabidopsis*, that loss of linker histone H1 affects the expression of known imprinted genes *MEA* and *FIS2*, encoding two essential components of the PRC2 complex, as well as *FWA*, encoding a transcription factor that controls flowering. Then, they observed that a loss of maternal H1 indirectly influences DEMETER-mediated DNA demethylation and chromatin architecture in the central cell and endosperm and suggested that H1-bound heterochromatin may act as a barrier to limit DEMETER DNA demethylation. Nevertheless, the loss of maternal H1 does not dramatically alter overall gene transcription and/or imprinting in the endosperm, and other selected imprinted genes presented variable dysregulation and methylation changes. Together, these results underline the extreme complexity of crosstalk between different epigenetic mechanisms, such as DNA methylation and chromatin accessibility to regulate gene expression.

Related to this work, Guo et al. describe the identification and characterization of linker histones in castor bean (*Ricinus communis*) in comparison to other economically important Euphorbiaceae species (*Hevea brasiliensis*, *Jatropha curcas*, *Manihot esculenta*, *Mercurialis annua*, and *Vernicia fordii*) and also the model plant *Arabidopsis thaliana*. Authors demonstrate how linker histones vary within and between populations and species and reveal their putative functions in castor bean and possibly more broadly, as many lines of possible future research projects.

In a last research article, Xiao et al., describe how they identified and cloned *TaDREB2B* from a wild species of sugarcane (*Tripidium arundinaceum*), a member of the DREB (Dehydration Responsive Element-Binding) subfamily transcription factor known to play a crucial role in plant drought response. Then, under the control of a drought-inducible promoter previously identified, authors expressed *TaDREB2B* in a commercial sugarcane cultivar and managed to improve its drought tolerance in the field by enhancing water retention capacity and reducing membrane damage, without affecting yield. Considering the ongoing climate change, drought-resistance is undoubtedly a key trait in crop breeding and studying epigenetic mechanisms involved in its regulation appears now essential for future crop improvements.

Besides these two research articles, the mini-review by Huang et al. focuses on how the transcriptionally corepressor HIGH EXPRESSION OF OSMOTICALLY RESPONSIVE GENE 15 (HOS15) epigenetically regulates flowering. In addition, authors highlight how HOS15 may contribute to integrate both endogenous and exogenous cues to establish an optimal trade-off between development and environmental adaptations.

Finally, Moebes et al. present a method enabling optimal RT-qPCR analyses of low-expression epigenetic genes in rapeseed with applications in stress response and crop improvement.

In conclusion, this Research Topic issue highlights the importance of diversify our models and our approaches in order to better understand how epigenetic changes contribute to modulate plant responses and adaptation in a changing environment.

## Author contributions

RA-V wrote the first draft of the article. AB made a substantial intellectual contribution to the work and approved it for publication.

